# Development and performance of a targeted whole exome sequencing enrichment kit for the dog (Canis Familiaris Build 3.1)

**DOI:** 10.1038/srep05597

**Published:** 2014-07-07

**Authors:** Bart J. G. Broeckx, Frank Coopman, Geert E. C. Verhoeven, Valérie Bavegems, Sarah De Keulenaer, Ellen De Meester, Filip Van Niewerburgh, Dieter Deforce

**Affiliations:** 1 Laboratory of Pharmaceutical Biotechnology, Faculty of Pharmaceutical Sciences, Ghent University, Ghent, Belgium; 2 Department of Applied Biosciences, Faculty of Biosciences Engineering, Ghent University, Ghent, Belgium; 3 Department of Medical Imaging and Small Animal Orthopaedics, Faculty of Veterinary Medicine, Ghent University, Merelbeke, Belgium; 4 Department of Medicine and Clinical Biology of Small Animals, Faculty of Veterinary Medicine, Ghent University, Merelbeke, Belgium; 5 These authors contributed equally to this work.

## Abstract

Whole exome sequencing is a technique that aims to selectively sequence all exons of protein-coding genes. A canine whole exome sequencing enrichment kit was designed based on the latest canine reference genome (build 3.1.72). Its performance was tested by sequencing 2 exome captures, each consisting of 4 pre-capture pooled, barcoded Illumina libraries on an Illumina HiSeq 2500. At an average sequencing depth of 102x, 83 to 86% of the target regions were completely sequenced with a minimum coverage of five and 90% of the reads mapped on the target regions. Additionally, it is shown that the reproducibility within and between captures is high and that pooling four samples per capture is a valid option. Overall, we have demonstrated the strong performance of this WES enrichment kit and are confident it will be a valuable tool in future disease association studies.

Since the first reported study on Whole Exome Sequencing (WES) in 2007^1^, well over 2000 papers have been published applying this technique (PubMed search: “exome sequencing”). WES is a cost-efficient approach to selectively sequence the coding regions of the genome. This approach allows to identify most functional variation without the high costs associated with whole-genome sequencing (WGS). Unfortunately, predesigned and validated kits are only commercially available for human and mouse. Scientific reports of WES on other animals are scarce^2^,^3^,^4^. This is unfortunate, as for example the dog is an excellent animal model for comparative disease genetics^5^. To fill the gap, we designed a canine exome sequencing kit (based on build 3.1.72) and tested its performance using Illumina Sequencing.

## Results

### Design

We designed a canine whole exome sequencing enrichment kit based on the latest canine reference genome (Broad CanFam 3.1.72)^6^. This design was based on the combination of the Ensembl Genes, the RefSeq Genes and the mRNA annotation. Additionally, known microRNAs were added from miRBase. After merging overlapping regions, the total size of the design was 52,876,195 bp (≈2% of the genome) divided over 203,059 regions. Based on our design, capturing baits were developed by Roche Nimblegen to target the specific regions. To avoid too much off-target sequencing, the most stringent setting was chosen for the baits design, allowing only unique matches from each bait to the reference genome.

### Performance: coverage and specificity

To assess the performance of the WES enrichtment kit, two exome captures were done, each consisting of 4 pooled samples. Sequencing depth, coverage of targeted regions and targeted bases and specificity were assessed for every sample. The results are reported for a minimum coverage of one and five (as five was the threshold used for variant calling). Each capture library was sequenced in one Illumina HiSeq 2500 lane. The number of raw reads generated per sample varied between 74,657,388 and 111,624,766. After quality trimming, mapping and duplicate read removal, between 87% and 90% of the reads were retained (Table 1). The average sequencing depth overall was 102x and ranged from 82.6x to 125.1x (Table 1).

**Figure 1:**
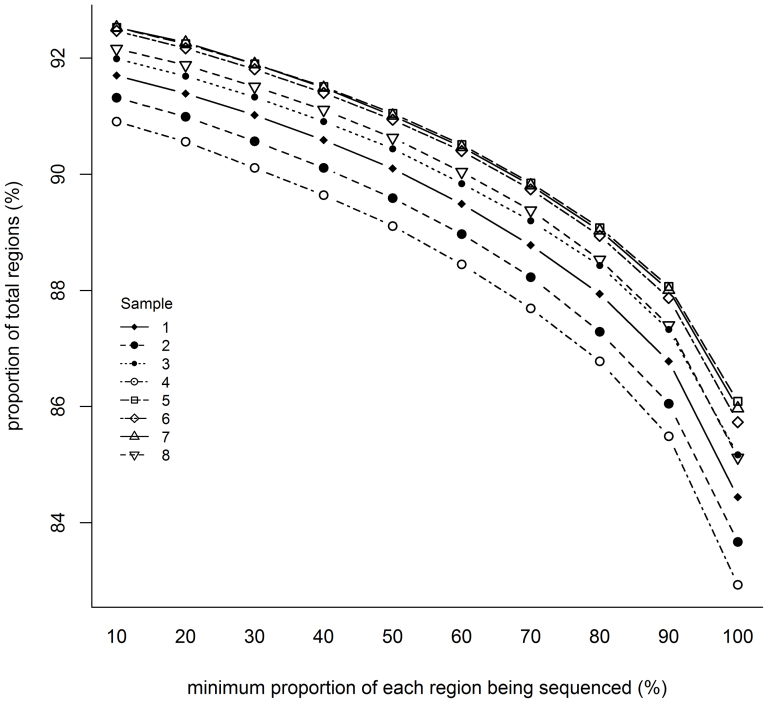
Relation between the proportion of each region being sequenced and the total amount of regions sequenced (%). For each individual region per sample, the percentage of the region being sequenced at a minimum coverage of 5, was calculated. On average 85% of the regions were completely sequenced. This number increased to 87% of the regions being sequenced for at least 90%. Around 90% of the regions were being sequenced for at least 60%.

Overall, an average of 92% of the regions were covered by at least one read and 90% by at least five reads. At a minimum coverage of one, 89 to 90% of the regions in our design, were completely covered. 83 to 86% of the regions were completely covered when a minimum coverage of five was applied (Figure 1). A clear relationship exists between the percentage of each region being sequenced and the proportion of total regions being sequenced (Figure 1). On average, a minimum coverage of 5 was not consistently reached throughout the entire region for 15% of the regions (Table 2). However, for only 8% of the regions on average, the maximum coverage never reached 5 (Table 2).

When looking at the coverage of targeted bases instead of regions, 93 to 94% (≈49 Mb) of the targeted bases (≈53 Mb) were covered at least once and 89 to 91% were covered at least five times (Table 3). We also assessed the specificity (reads on target/total number of reads). With an average overall specificity of 90%, off-target sequencing is rather small and comparable with earlier reports^7^. The specificity was also assessed in every single sample per chromosome. For all eight samples, results were similar, with the highest specificity on average found on chromosome nine (94.05%) and the lowest specificity found on average on chromosome 22 (84.09%). Per chromosome specificity is available in Supplementary Table S1.

### Performance: reproducibility

The reproducibility within and between captures was checked by comparing the amount of targeted bases and regions that are sequenced at least once and five times in every single sample. Overall, from the ≈53 Mb target base pairs, 48,141,464 base pairs (91.0%) were sequenced at least once in all eight samples. We also assessed how many base pairs were never sequenced. Overall, 2,313,892 base pairs (4.4%) were never covered. Overall, the remaining 4.6% of the total target base pairs are being sequenced variably. Comparing the 4 samples within each capture, we found that 48,333,432 (91.4%) or 48,663,244 (92.0%) base pairs were common and 2,816,548 (5.3%) or 2,553,759 (4.8%) base pairs were never sequenced. A similar analysis was conducted for a coverage of 5. For all eight samples, 46,236,131 base pairs (87.4%) were sequenced consistently with a minimum coverage of 5 and 4,078,886 base pairs (7.7%) never reached a coverage of 5. 46,439,217 (87.8%) or 47,102,104 (89.1%) and 4,572,396 (8.6%) or 4,216,408 (8.0%) base pairs were common within each pool reaching a coverage of at least 5 or never reaching a coverage of 5, respectively.

The regions in common were also assessed for a coverage of 1 and 5. From the 203,059 regions, overall, 4,791 (2.4%) regions were never sequenced and 176,645 (87,0%) were consistently covered at least once. Within each pool, 177,664 (87.5%) or 179,463 (88.4%) regions were common and 6,620 (3.3%) or 5,722 (2.8%) regions were never sequenced. For a coverage of 5, 11,691 regions (5.8%) were consistently not sequenced sufficiently and 160,366 (79.0%) regions were. This results in 31,002 (15.3%) of the regions being variably sequenced. Within each pool for a coverage of 5, 162,312 (79.9%) or 167,830 (82.7%) reqions were sequenced and 13,484 (6.6%) or 12192 (6.0%) regions were not. The non-covered base pairs and regions are probably a consequence of the chosen stringency when baits were designed as only unique matches were allowed. A table containing these annotated 11,691 regions is available on request.

### Sample pooling

Pooling several samples together prior to capturing is common practice, mainly to reduce cost. Of course, pre-capture pooling should only be done when it does not significantly decrease the enrichment performance. To check the effect of pre-capture pooling, we created subsets containing 25% randomly chosen reads out of the total number of reads in the combined output of the 4 samples per capture. The rationale is to simulate samples as if they were not barcoded and as if the DNA strands presented to the capture baits are from one sample. A random subset of 25% needs to be taken to reduce the total number of reads to a number comparable to the number of reads in the individual barcoded samples. Per pool, ten subsets were created, resulting in a total of twenty new samples. Comparing the number of regions that are completely covered at least once in the subsets and the original samples, an average of 687 and 684 additional regions (≈0.3%) were covered in the subsets of pool one and two, respectively. An average of 119,097 and 131,774 additional target base pairs were covered at least once in the respective subsets, which represents 0.2% of the ≈53 Mb design. At a minimum sequencing depth of 5, an average of 1,705 (0.8%) and 1,468 (0.7%) additional completely covered regions and an additional 174,070 (0.3%) and 164,522 (0.3%) base pairs were covered for pool one and two, respectively. The average specificity increased from 90.85% to 91.48% and from 89.86% to 90.73% in both pools, respectively. Based on these results, we conclude that pre-capture pooling of samples is a valid option as the effect on the different performance parameters is minimal. The exact number of samples that can be pooled, depends on a cost-benefit assessment.

### Variant calling

Finally, we also called variants using a probabilistic variant caller. The number of non-reference variants called per sample, ranged from 55,683 to 60,576 and from 62,117 to 67,890 with the “require presence in both forward and reverse reads” setting being applied or not, respectively (Supplementary Table S2). Applying this setting might exclude variants at the boundaries of the targeted regions, however it decreases the amount of erroneous variants^8^.

## Discussion

This study is the first to report on the performance of an exome kit designed for the dog on the latest annotation (CanFam 3.1.72). With on average 90% of the regions and 90% of the bases covered five times, a high amount of the targeted regions is captured, without too much off-target sequencing as the specificity is 90%. The reproducibility within and between captures is high. Additionally, the results indicate that pooling four samples per capture is a valid option as it has only a very limited effect on the performance, but substantially reduces the costs. This makes WES even more affordable (compared with WGS). Finally, it is demonstrated that WES is capable to detect variants within the coding regions. Overall, we have demonstrated the strong performance of this WES enrichment kit and are confident it will be a valuable tool in future disease association studies.

## Methods

### Sample collection

Eight blood samples were obtained from a canine blood bank available at Ghent University to study genetic disorders^9^. Approval was granted by the local ethical (Faculty of Veterinary Medicine, Ghent University, Belgium) and deontological (Federal Public Service Health, Food Chain Safety and Environment, Brussels, Belgium) committees (EC2013_193).

### Design

The data needed to design the exome kit was downloaded from the University Of California Santa Cruz (UCSC) (http://genome.ucsc.edu/) table browser (Dog, CanFam3.1)^10^. From the Genes and Gene prediction tracks, RefSeq Genes and Ensembl Genes were selected. The output format was a BED file with the setting “exons (plus 0 bases at each end)”. From the mRNA and EST Tracks, Dog mRNAs and all_mrna were selected respectively. The output format was also a BED file with the “blocks plus 0 bases at each end” setting. Micro RNA sequence positions were downloaded from miRBase^11^. Regions were merged using bedtools version v2.17.0. The total size of the design was 52,876,195 Mb (≈2% of the genome) divided over 203,059 regions. The BED file is available on request.

### Roche Nimblegen WES enrichment kit

Our design was processed by the Roche Nimblegen custom design group (Madison, USA). Using an SSAHA algorithm, capturing baits were developed based on our design and the reference genome of the dog (Canis Familiaris 3.1). Design settings for the baits allowed five or fewer single-base insertions, deletions or substitutions between the baits and the genome. Each bait itself was only allowed to match one location in the genome to avoid too much off target sequencing. Regions under 100 bp were padded to 100 bp to increase capturing efficiency. After approval, the baits were generated and provided as SeqCap Developer Library.

### DNA extraction

Genomic DNA was extracted with the DNeasy Blood & Tissue Kit (QIAGEN) with 100 µl of blood as input. The standard protocol was followed with the exception of the final elution step: instead of using 200 µl of Buffer AE, only 50 µl was used. The eluate was used again to elute a second and third time to increase the concentration. The DNA yield was measured with Quant-iT™ Picogreen® dsDNA Assay (Life Technologies).

### Sample preparation and sequencing

Extracted DNA was fragmented on a Covaris S2 System in a 50 µl volume (aim: 300 bp fragments, settings: duty cycle: 10%, intensity: 5, cycles per burst: 200, time: 50 s). After shearing, another picogreen assay was performed. Around one µg of the fragmented DNA was used as input for the library preparation. Samples were end repaired, A-tailed and ligated with TruSeq adapters using the reagents from the NEBNext DNA Library Prep Master mix set for Illumina (New England Biolabs) according to the manufacturer's protocol. AMPure XP beads (Beckman Coulter) were used for selection of fragments with an insert size around 300 bp. One µl of the ligated product was subsequently amplified in an enrichment PCR (10 cycles) for library quality assessment as recommended in the ‘SeqCap EZ Library SR User's Guide’ (Nimblegen, Roche). Thereafter, the pre-capture LM-PCR was performed on the samples for 8 cycles as prescribed in the SeqCap EZ library protocol. The concentration of each PCR product was determined using Quant-iT^TM^ Picogreen® dsDNA Assay (Life Technologies). Two times four samples were equimolarly pooled to obtain a total DNA input of 1250 ng. The pooled library was hybridized for 67–68 hours with the baits (SeqCap Developer Library). The hybridized library was washed and the captured and pooled DNA was recovered. After a final amplification (LM-PCR, 18 cycles), the quality of the library was checked using the High Sensitivity DNA chip (Agilent).

### QPCR

To check the fold enrichment after capturing, a qPCR is performed as a final quality control step before sequencing. We chose to test five loci. Primer one is the standard primer provided by Roche Nimblegen (NSC-0237). The other four primers were designed using NCBI Primer-BLAST (http://www.ncbi.nlm.nih.gov/tools/primer-blast/)^12^. Sequences are available in Supplementary Table S3. The amplification efficiency of each primer was determined by qPCR. One µl of the following template DNA quantities were added to each reaction: 20 ng, 10 ng, 5 ng, 2.5 ng and 1.25 ng. Each reaction was performed in triplicate. Efficiencies E were calculated with the following formula: E = 10^(–1/slope of standard curve)^ and are mentioned in Supplementary Table S3. To assess the fold enrichment for both pools of four samples, a qPCR was performed according to the instructions from Roche Nimblegen. Fold enrichment was calculated using the following formula: (E^delta-Ct^) with delta-Ct being the difference in threshold cycle between the library prior and post capturing. The average fold enrichment was well over the tenfold threshold suggested by Roche Nimblegen.

### Sequencing

The two pools were sequenced on two different lanes in two different runs on a HiSeq 2500 PE 100 bp.

### Data-analysis

Data-analysis was performed using the CLC Genomics Workbench (Version 6.5.1, CLC Bio, Aarhus, Denmark). Data was trimmed with the following settings: ambiguous trim = no, quality trim = yes, quality limit = 0.05, use colorspace = no, create report = yes, also search on reversed sequence = yes, save discarded sequences = yes, remove 5′ terminal nucleotides = no, discard short reads = no, remove 3′ terminal nucleotides = no, trim adapter list = adapter list Illumina, discard long reads = no, save broken pairs = yes. The reference genome was downloaded from the UCSC genome browser^6^. For read mapping, the following parameters were used: mismatch cost = 2, insertion and deletion cost = 3, length fraction: 0.5, similarity fraction = 0.8, global alignment = no, auto-detect paired distances = yes, non-specific match handling = ignore, output mode = create reads track, create report = yes, collect un-mapped reads = yes. Duplicated reads were removed with the Duplicate Mapped Reads Removal (Version 1.0 beta 5) plugin (setting: maximum representation of minority sequence (percent) to 20.0). Reads were locally realigned with the following settings: realign unaligned ends = yes, multi-pass realignment = 3, guidance-variant track = not set, output mode = create reads track, output track of realigned regions = yes. Variants were called twice using probability variant detection with the following settings: ignore non-specific matches = yes, ignore broken pairs = yes, minimum coverage = 5, variant probability = 90.0, required variant count = 2, ignore variants in non-specific regions = yes, filter 454/Ion homopolymer indels = no, maximum expected variants = 2, genetic code = 1 standard, create track = yes, create annotated table = yes. The first variants were called with the “require presence in both forward and reverse reads = yes”, the second call was run without this setting.

### Effect of pooling

Forward and reverse reads from each pool were combined in two large pools. From each pool, ten random subsets were created using seqtk version 1.0-r31. Data was analysed using the same settings as the real samples.

## Author Contributions

Conceived and designed the experiments: B.B., F.V.N., D.D. Performed the experiments: B.B., V.B., S.D.K., E.D.M. Analyzed the data: B.B., F.V.N. Contributed reagents/materials/analysis tools: B.B., F.C., G.V., V.B., S.D.K., E.D.M., F.V.N., D.D. Wrote the paper: B.B., F.C., G.V., V.B., S.D.K., E.D.M., F.V.N., D.D.

## Additional information

**Accession code.** Exome sequencing data are available at the Sequence Read Archive under accession number PRJEB5259.

## Supplementary Material

Supplementary Information Supplementary material

## Figures and Tables

**Table 1:** Statistics for exome sequencing eight dogs

	Sequencing reads					
Sample	Total	Mapped	Duplicate	Remaining	Remaining (%)	Sequencing depth (x)
1	82,574,410	77,392,469	4,820,648	72,571,821	87.9	93.0
2	74,657,388	69,542,653	4,518,820	65,023,833	87.1	82.6
3	90,534,096	83,841,822	4,680,806	79,161,016	87.4	102.0
4	77,786,110	72,147,586	4,457,341	67,690,245	87.0	87.1
5	111,624,766	108,781,536	9,882,797	98,898,739	88.6	125.1
6	96,041,166	93,261,066	8,278,081	84,982,985	88.5	106.9
7	103,290,412	100,440,603	8,653,736	91,786,867	88.9	116.7
8	86,094,438	83,226,207	5,926,249	77,299,958	89.8	99.3

**Table 2:** Regions with a coverage below 5

Sample	Regions with minimum coverage <5 (%)	Regions with maximum coverage <5 (%)
1	31,604 (15.56)	16,330 (8.04)
2	33,167 (16.33)	17,042 (8.39)
3	30,122 (14.83)	15,800 (7.78)
4	34,655 (17.07)	17,831 (8.78)
5	28,250 (13.91)	14,733 (7.26)
6	28,979 (14.27)	14,824 (7.30)
7	28,487 (14.03)	14,696 (7.24)
8	30,224 (14.88)	15,465 (7.62)

**Table 3:** Coverage of targeted base pairs

Sample	% of target bp covered (>1x)	% of target bp covered (>5x)
1	93.15	89.96
2	92.90	89.54
3	93.22	90.24
4	92.82	89.15
5	93.52	90.63
6	93.66	90.71
7	93.53	90.63
8	93.56	90.53

The second and third column show the percentage of base pairs from the design of 52,876,195 basepairs with a coverage of at least one and five, respectively, within each sample.
