# Author Correction: Development and performance of a targeted whole exome sequencing enrichment kit for the dog (Canis Familiaris Build 3.1)

**DOI:** 10.1038/s41598-020-62785-3

**Published:** 2020-04-08

**Authors:** Bart J. G. Broeckx, Frank Coopman, Geert E. C. Verhoeven, Valérie Bavegems, Sarah De Keulenaer, Ellen De Meester, Filip Van Nieuwerburgh, Dieter Deforce

**Affiliations:** 1 Laboratory of Pharmaceutical Biotechnology, Faculty of Pharmaceutical Sciences, Ghent University, Ghent, Belgium; 2 Department of Applied Biosciences, Faculty of Biosciences Engineering, Ghent University, Ghent, Belgium; 3 Department of Medical Imaging and Small Animal Orthopaedics, Faculty of Veterinary Medicine, Ghent University, Merelbeke, Belgium; 4 Department of Medicine and Clinical Biology of Small Animals, Faculty of Veterinary Medicine, Ghent University, Merelbeke, Belgium

Correction to: *Scientific Reports* 10.1038/srep05597, published online 07 July 2014

This Article contains a typographical error in the spelling of the author Filip Van Nieuwerburgh, which is incorrectly given as Filip Van Niewerburgh.

