# Supplementary material for: Development and performance of a targeted whole exome sequencing enrichment kit for the dog (Canis Familiaris Build 3.1)
Source: Sci Rep. 2014 Jul 7;4:5597. doi: 10.1038/srep05597 (PMC4083258; doi:10.1038/srep05597)
Supplement: Supplementary Information — Supplementary material [file srep05597-s1.pdf]

Development and performance of a targeted whole exome sequencing enrichment kit for the dog (Canis Familiaris Build 3.1)

Bart JG Broeckx[1], Frank Coopman[2], Geert EC Verhoeven[3], Valérie Bavegems[4], Sarah De Keulenaer[1], Ellen De Meester[1], Filip Van Niewerburgh[1‡], Dieter Deforce[1‡*]

[1] Laboratory of Pharmaceutical Biotechnology, Faculty of Pharmaceutical Sciences, Ghent University, Ghent, Belgium

[2] Department of Applied Biosciences, Faculty of Biosciences Engineering, Ghent University, Ghent, Belgium

[3] Department of Medical Imaging and Small Animal Orthopaedics, Faculty of Veterinary Medicine, Ghent University, Merelbeke, Belgium

[4] Department of Medicine and Clinical Biology of Small Animals, Faculty of Veterinary Medicine, Ghent University, Merelbeke, Belgium

‡ These authors contributed equally to this work.

Supplementary Table S1 Per sample per chromosome specificity (calculated as mapped reads on target/total number of mapped reads)

	Specificity per sample (%)							
Chr	1	2	3	4	5	6	7	8
1	92.23	91.96	92.57	92.26	91.65	91.3	91.20	91.79
2	91.59	91.29	92.02	91.71	91.01	90.66	90.52	91.10
3	89.48	89.41	89.96	89.78	88.43	88.17	87.83	88.59
4	90.26	90.12	90.64	90.53	89.43	89.05	88.75	89.46
5	92.82	92.55	93.17	92.8	92.32	91.91	91.82	92.38
6	92.68	92.46	93.13	92.74	92.26	91.8	91.75	92.44
7	91.95	91.67	92.24	92.13	91.37	90.92	90.82	91.39
8	90.28	90.10	90.80	90.44	89.63	89.31	89.12	89.86
9	94.19	93.88	94.53	94.17	94.22	93.66	93.66	94.11
10	91.45	91.26	91.88	91.61	90.84	90.49	90.36	90.96
11	91.06	90.88	91.36	91.19	90.43	90.07	89.88	90.46
12	91.73	91.53	92.16	91.89	91.17	90.86	90.69	91.35
13	89.63	89.63	90.18	90.14	88.60	88.50	88.09	88.80
14	89.98	89.81	90.37	90.29	88.99	88.75	88.42	89.05
15	91.03	90.83	91.47	91.28	90.31	90.01	89.80	90.40
16	89.82	89.33	90.31	89.89	88.66	88.59	88.71	89.20
17	91.99	91.74	92.38	92.12	91.50	91.07	91.00	91.50
18	92.91	92.63	93.28	92.80	92.44	92.12	92.07	92.66
19	85.92	86.31	86.69	86.73	84.87	84.63	83.91	84.78
20	93.36	93.01	93.69	93.12	93.12	92.70	92.74	93.30
21	91.15	90.93	91.46	91.12	90.43	90.09	90.03	90.54
22	84.69	84.86	85.38	85.35	83.18	83.21	82.59	83.44
23	90.41	90.34	90.78	90.77	89.64	89.27	88.96	89.58
24	92.08	91.89	92.44	92.05	91.55	91.14	91.06	91.68
25	90.81	90.68	91.29	91.11	90.07	89.85	89.62	90.25
26	92.22	91.96	92.55	92.19	91.95	91.53	91.34	92.02
27	92.33	92.01	92.65	92.37	91.98	91.54	91.50	92.06
28	91.64	91.47	92.02	91.93	91.26	90.77	90.60	91.03
29	88.01	87.98	88.57	88.75	86.75	86.59	85.97	86.70
30	92.91	92.66	93.22	93.06	92.70	92.28	92.03	92.57
31	86.13	86.18	86.75	86.23	84.99	84.57	84.36	85.40
32	88.97	88.81	89.37	89.57	88.30	88.08	87.72	88.19
33	91.77	91.67	92.05	92.13	91.20	90.76	90.61	91.05
34	88.89	88.71	89.31	89.08	87.74	87.52	87.12	87.96
35	89.14	89.03	89.53	89.41	88.07	87.84	87.42	88.21
36	92.80	92.67	93.20	93.30	92.20	91.95	91.73	92.04
37	91.20	91.10	91.62	91.57	90.66	90.26	90.00	90.58
38	91.20	90.98	91.70	91.16	90.46	90.16	89.97	90.63
X	87.85	87.88	88.53	87.99	87.74	87.62	87.26	87.89

Supplementary Table S2 Non-reference variants detected on target per sample

Sample	Variants (bidirectional)	Variants
1	57,827	65,060
2	56,401	63,491
3	58,769	66,639
4	57,623	64,793
5	60,444	67,222
6	60,576	67,890
7	55,683	62,117
8	58,615	65,967

The second and third column show the number of variants being called with and without "the "require presence in both forward and reverse reads" setting being applied, respectively.

Supplementary Table S3 Primers (and their efficiencies) used to assess fold enrichment

Primer	Forward	Reverse	E	Chr
1	5'-CGCATTCCTCATCCCAGTATG-3'	5'-AAAGGACTTGGTGCAGAGTTCAG-3'	1.60	12
2	5'-GTAGTGAGGCGAGTGGCTTT-3'	5'-CCGACAGCACTACATGGGTT-3'	2.06	36
3	5'-CTCCTGGGGCACAAATGAGT-3'	5'-AGGGAGAATATGGCCCACCT-3'	1.81	30
4	5'-TCTGTGAGGGTGGCTTTTCC-3'	5'-TCTCTGGGGCATCTGTGAGA-3'	1.75	17
5	5'-TCGCTGACGTGTTCAAAGGA-3'	5'-AGAACCCACGCCTGAAGATG-3'	1.67	3

The second and third column contain the sequences of all five primers. Primer one is the standard primer provided by Nimblegen (NSC-0237). The fourth column contains the efficiency of amplification (E) as calculated with the following formula: $E = 10^{(-1/\text{slope of standard curve})}$. The chromosome on which the control locus is located is mentioned in the fifth column.
